# Multiway PCA for Early Leak Detection in a Pipeline System of a Steam Boiler—Selected Case Studies

**DOI:** 10.3390/s20061561

**Published:** 2020-03-11

**Authors:** Miroslaw Swiercz, Halina Mroczkowska

**Affiliations:** 1 Faculty of Electrical Engineering, Bialystok University of Technology, Wiejska 45D, 15-351 Bialystok, Poland; 2 Enea Cieplo sp. z o.o., ul. Warszawska 27, 15-062 Bialystok, Poland; halina.mroczkowska@enea.pl

**Keywords:** fault detection, pipeline leaks, steam boiler, multiway PCA method

## Abstract

In the paper the usability of the Multiway PCA (MPCA) method for early detection of leakages in the pipeline system of a steam boiler in a thermal-electrical power plant is presented. A long segment of measurements of selected process variables was divided into a series of “batches” (representing daily recordings of normal behavior of the plant) and used to create the MPCA model of a “healthy” system in a reduced space of three principal components (PC). The periodically updated MPCA model was used to establish the confidence ellipsoid for the “healthy” system in the PC coordinates. The staff’s decision of the probable leak detection is supported by comparison of the current location of the operating point (on the “fault trajectory”) with the boundaries of the confidence ellipsoid. It must be emphasized that due to daily and seasonal changes of heat/electricity demands, the process variables have substantially greater variability than in the examples of batch processes studied in literature. Despite those real challenges for the MPCA method, numerical examples confirmed that the presented approach was able to foresee the leaks earlier than the operator, typically 3–5 days before the boiler shutdown. The presented methodology may be useful in implementation of an on-line system, developed to improve safety and maintenance of boilers in a thermal-electrical power plant.

## 1. Introduction

Due to increasing complexity and costs of modern manufacturing processes, plants must be equipped both with efficient control systems and the tools for on-line fault detection and diagnosis of a production process. The faults of technological components; faults of measurement and control devices; and staff errors can cause serious material or human losses, so the fault detection and identification system should provide acceptable level of reliability and guarantee the safety of the technical personnel. An automatic fault detection system, working together with process control, should be of course the ideal solution; however, in the case of most industrial processes, various semiautomatic tools which warn process operators against developing failures are considered satisfactory. The crucial requirement in the design and implementation of the fault detection system is its ability to discover the fault symptoms as early as possible, to give the staff enough time to change the control policy; repair the faulty device; or eventually, to shut down the process in a safe manner.

Fault diagnosis methods can be based on the following approaches [1]:

- Signal processing, when spectral analysis, principal component analysis (PCA), wavelet transforms, fast Fourier transforms (FFTs), etc., are used to analyze the system and identify faults;
- Model-based methodologies, when knowledge of the system (in the form of physical, balance and chemical equations; or a black-box or a grey-box model) is employed to detect and analyze faults;
- Artificial intelligence, when neural networks, fuzzy systems, expert systems, gray correlation or support vector machines (SVMs) are used to develop a diagnostic system that, once trained, can identify specific faults.

Boilers are important components in power, chemical and oil refinery industries; they transform water into steam for power generation or other industrial applications. A common boiler fault is the tube leak in the riser and downcomer sections due to aging (corrosion) and thermal stress (e.g., overheating). Early detection of such faults in operation is important, because it helps in reducing possible damage to equipment and productivity loss caused by (otherwise) unscheduled boiler shutdown, and also ensures safety for operators [2]. Several methods of leakage detection in a boiler pipeline system have been described in literature; however, some of them cannot be easily applied in industrial practice, especially in a plant which was designed and equipped over fifteen years ago and has been working under certain technical and economical conditions. That is why we used an approach from the group of statistical process control (SPC) methods for fault detection in a real thermal-electrical power plant. The methodology described below uses historical data recorded by the measurement and control system to extract symptoms of a developing failure and give the process operator clear warning in a simple, comprehensible way.

Briefly, the paper is organized as follows. Section 2 presents a short overview of boiler leak detection methods, currently used in industrial practice or developed and verified on laboratory equipment. In that section, several examples of the MPCA method applied to the boiler fault detection problem are also presented. Section 3 focuses on theoretical background of the principal component analysis (PCA) algorithm and its modification—the multiway PCA method. In Section 4, the boiler water system working in Elektrocieplownia Bialystok is briefly presented. The problem statement of leakage detection in a steam boiler pipeline system in Elektrocieplownia Bialystok; analysis of the available process data; and presentation and discussion of two case studies are the contents of Section 5. Finally, Section 6 offers some concluding remarks and considerations about further work on boiler leakage detection in Elektrocieplownia Bialystok.

## 2. Current Approaches to Fault Detection in a Pipeline System of a Steam Boiler

Boilers are complex, nonlinear systems, which work under time-varying operating conditions due to daily and seasonal changes of heat/electricity demands. The complexity of physical processes which take place in a boiler, their actual nonlinearity, various characteristics of process disturbances (e.g., fluctuations in the combustion process) and technical limitations of acquisition data necessary for identification of mathematical models in every stage of the process make the modeling task truly difficult. That is why mathematical models potentially useful for pipeline leak detection usually have the form of the black-box or simple linear models [3], and only a small class of models, which are based on physical relationships, may correspond in a limited scope to a certain industrial boiler.

The leakage in a boiler pipeline system is a special type of a process fault, whose development may be considered as a non-stationary, nonlinear process. At its early stage, such a fault may be compensated (and “masked”) by normal control actions, such as those carried out in a standard control response to process disturbances. However, early detection of small leaks in the pipelines can protect the plant against secondary malfunctions or damages and unscheduled shutdowns, and provide improved safety.

Detection and localization of leaks in a pipeline system of an industrial steam boiler is a difficult problem, studied in a limited number of research papers. Due to small loses of mass and energy per time unit, the pipe leaks at their early stages can hardly be detected with the use of simple methods of limitation checking or statistical analysis, or the steady-state based methods. The methods of leak detection in pipelines of steam boilers, which are most frequently used in industrial practice, can be roughly divided into the following groups [4]:

1. Acoustic monitoring and analysis, which uses acoustic waves generated by the escaping steam—the methods require installation of rather expensive devices (sensors) and careful tuning; they cannot detect small to medium leaks (less than about 10,000 kg/h);
2. Steam/water balance testing, which is rather time consuming and insensitive to small leaks; usually, the maximal frequency of tests is too low for preventing serious damage to the pipeline system;
3. Monitoring of gas humidity in the flue, which has limited specificity, as the measured changes of humidity can be caused by water added to the combustion chamber, soot blowing, etc., and by steam leaks to be detected;
4. Other methods, based on monitoring and analysis of process variables and their relationships, sometimes supported by a mathematical model of the technological process.

In the acoustic leak monitoring systems, the turbulence caused by the fluid escaping from a leak in a boiler tube generates high frequency pressure waves (airborne and structure-borne acoustic waves) within the contained fluid itself, throughout the flue gas into which the fluid is escaping, and within the container structure. The energy associated with these mechanical waves is converted into electronic voltage signals with a variety of sensitive dynamic pressure transducers (sensors). The multi-channel signals are amplified, filtered and processed to determine energy content [5,6], and then continuously analyzed with the use of software tools to detect abnormalities corresponding to leaks, and if possible, localize the probable place where the fault occurred [7,8,9]. However the acoustic leak detection systems that cannot detect small and medium leaks of the typical water loss < 10,000 kg/h, are quite expensive and require advanced signal processing and analysis tools to compare the characteristics of the “acoustic scene” in normal operating conditions with the features of signals recorded in abnormal conditions. The changes of the plant operating point cause substantial variations in the characteristics of the signal (e.g., its power spectral density) and the contents of the process noise (e.g., due to geometry of the furnace, generating echoes) influence the results of signal processing and makes the decision task very complicated.

Detecting leaks using water or chemical mass balance methods around a boiler is quite simple, as flow meters around the waterside of the boiler may be used to calculate the amount of water and nonvolatile species (such as phosphate or molybdate) entering and leaving the boiler. If a statistically significant loss is calculated, then a water leak is suspected [10]. The main reason for inaccuracy of the mass balance methods is the presence of variations in the individual and composite signals, which are regarded as the process noise. Other serious drawbacks of the method are that it cannot be used for localization of the leakage and is insensitive to small leaks (which take place, e.g., at the early stage of development of a tube crack). Additionally, the methods of mass balance testing are time consuming and are performed with a frequency which is not sufficient to protect the plant from serious damage. More advanced approaches to leak detection by the use of mass balance testing are based on the Input/Loss Method, a patented method (based on integration of system stoichiometrics with thermodynamics) which computes fuel chemistry, heating value and fuel flow by integrating effluent measurements with thermodynamics [11].

Several other interesting approaches to leak detection in steam boilers, presented in the literature in the last few years, were implemented in industrial practice, performed on small or medium-size pilot plants, equipped with measurement devices typically used in industry, or based on the processing of real data recorded in heat and electricity plants in simulation experiments. Some of the approaches utilize different kinds of mathematical models of the process and sophisticated tools for signal processing and classification (e.g., neural networks, fuzzy systems, genetic algorithms and other AI tools). In the model-based approach to fault detection, models which describe input-output relationships in a boiler working in normal and abnormal (faulty) conditions are built and identified, based on real data [12]. The data recorded from a real plant are then compared with the model outputs (using, e.g., the least-squares algorithm) to detect the leakage. Some approaches [13,14] employ state estimators and observers to detect faults by tracking the dynamics of errors in the presence of model uncertainties (e.g., caused by process faults).

The artificial neural networks (ANNs), as universal approximators of any nonlinear input-output mappings, have been used in advanced control and fault detection schemes, both as process models and as nonlinear controllers [15]. The ANNs confirmed their ability to utilize real-time data taken from a running boiler system and periodically adapt to changeable process characteristics [16,17]. Neural networks are also combined with fuzzy logic, both for modeling of a boiler system and for detection of process faults [18]. A combination of fuzzy logic, neural networks and genetic algorithms (GAs) was employed to develop proper models for the subsystems of a steam boiler in [19]. Each subsystem was considered as a concise multilayer neuro-fuzzy model, while GAs were applied to extract the optimized fuzzy rules for each subsystem.

The efficiency of the methods based on mathematical models strongly depends on the model adequacy, as well as on the precision and accuracy of parameter identification. The alternative, data driven methods (for example, statistical dimensionality reduction), find patterns corresponding to normal operating conditions and faulty conditions or compute meaningful statistics directly from the process historical data. Such an approach eliminates the use of detailed models of large-scale systems, what can be expensive and difficult to develop [20]. From this group of models, principal component analysis (PCA) and its modified versions have been also applied to detection of different faults in steam boilers. Some interesting extensions of the basic PCA method have been proposed in literature; e.g., the moving cumulative alarm (MCA) technique [21]—a data preprocessing scheme which can reduce the negative influence of noise and disturbances on boiler leak detection, and thus can improve the detectability of faults for Hotelling’s $T^{2}$ and squared prediction error (SPE) statistics. Furthermore, additional statistics, e.g., the prediction residual sum of squares (PRESS) statistic [22], have been proposed together with commonly used $T^{2}$ and Q statistics to evaluate matching of data to the PCA model of a normal (not-faulty) system.

One of the main reasons for the lower than expected efficiency of the PCA method is the relatively frequent occurrence of changes in the operating conditions of the boiler. In such a situation, it is difficult to describe the statistical properties of the process with a single principal component model (PCM), and using a traditional PCA-based fault detection method can bring many misdiagnoses. The approach proposed by some authors [23] consists of establishing a group of PCMs—each of them corresponds to one stable operating point and is built on the basis of a data subset separated from the entire process data with the use of cluster analysis. The newly acquired data sample is then assigned to the “nearest” model with the use of a suitable classifier.

## 3. Methodology of Principal Component Analysis and Multiway Principal Component Analysis

### 3.1. Principal Component Analysis—Theoretical Preliminaries

Principal component analysis (PCA) is an unsupervised, linear multivariate statistic technique of dimensionality reduction; decorrelation; and to some extent, denoising of a set of data, obtained during process monitoring. PCA transforms a set of correlated variables with the zero mean value into a new set of latent variables called principal components (PCs). The principal components, which are linear combinations of the original variables, are decorrelated and mutually orthogonal. The first principal component (PC1) defines the direction of the greatest variability within the original data set, with subsequent principal components explaining a decreased amount of variability. Consequently, lower order principal components can be excluded without losing essential information from the original variables, as they characterize mainly process noise. By retaining a limited number of principal components, the use of the PCA method reduces the problem dimensionality and extracts the features of the data set [24].

Data to be decomposed by the PCA method are gathered in a matrix $X\in R^{n\times m}$, which consists of *m* variables and *n* samples (observations) as below:

(1) $$X=[\begin{array}{cc} \begin{array}{cc} x_{11} & x_{12}\\ x_{21} & x_{22} \end{array}\; & \begin{array}{cc} \cdots & x_{1m}\\ \cdots & \cdots \end{array}\\ \begin{array}{cc} \cdots & \cdots \\ x_{n1} & x_{n2} \end{array}\; & \begin{array}{cc} \cdots & x_{n-1,m}\\ \cdots & x_{nm} \end{array} \end{array}]$$

The mean value of each variable and the covariance of two variables can be computed as:

(2) $${\bar{x}}_{i}=\frac{1}{n}\overset{n}{\underset{k=1}{\sum }}x_{ki}\;\;\;for\;i=1\cdots m$$

(3) $$q_{ij}=\frac{1}{n-1}\sum_{k=1}^{n}(x_{ki}-{\bar{x}}_{i})(x_{kj}-{\bar{x}}_{j})\;\;\;for\;i=1\cdots mandj=1\cdots m$$

Assuming that each column of the matrix ***X*** (which contains a data vector) is centered about its mean and scaled to unity variance, the empirical covariance matrix is expressed by:

(4) $$S=\frac{1}{n-1}X^{T}X$$

Using a singular value decomposition of the matrix ***X***, Equation (4) can be expressed as:

(5) $$S=\frac{1}{n-1}X^{T}X\;=\;{\left(U\Sigma V^{T}\right)}^{T}\times (U\Sigma V^{T})\;=\;V\Lambda V^{T}$$

where $\Lambda =\Sigma^{T}\Sigma \in R^{m\times m}$ is a diagonal matrix with nonnegative elements of its main diagonal, while $U\in R^{n\times n}$ and $V\in R^{m\times m}$ are unitary matrices, i.e.,

(6) $$U^{T}U\;=\;UU^{T}\;=\;I\;\;\;\;\;and\;\;\;\;\;V^{T}V\;=\;VV^{T}\;=\;I$$

The elements on the main diagonal of the matrix Λ are real eigenvalues in not increasing order (i.e., $\lambda_{1}\geq \lambda_{2}\geq \cdots \geq \lambda_{m}\geq 0$), and the *i*-th eigenvalue is the square of the *i*-th singular value: $\lambda_{i}=\sigma_{i}^{2}$. The diagonal matrix Λ is the covariance matrix of the principal components and consists of eigenvalues of the covariance matrix ***S***.

PCA decomposes the measurement matrix ***X*** by the projection onto two orthogonal latent subspaces—one is the principal component subspace ($\hat{X}$), capturing the most of data variations, and the other is the residual subspace ($\tilde{X}$) that includes some uncorrelated changes and noises [25]. Therefore, the decomposition of the matrix ***X*** can be presented as a sum of the outer product of vectors $t_{i}$ and $p_{i}$, as follows:

(7) $$X\;=\overset{l}{\underset{i=1}{\sum }}t_{i}P_{i}^{T}+\overset{m-l}{\underset{i=1}{\sum }}{\tilde{t}}_{i}{\tilde{P}}_{i}^{T}$$

or

(8) $$X=\hat{X}+\tilde{X}=TP^{T}+E=TP^{T}+\tilde{T}{\tilde{P}}^{T}=[T|\tilde{T}]{\left[P|\tilde{P}\right]}^{T}=\bar{T}{\bar{P}}^{T}$$

where the matrices ***T*** and ***P*** stand for the score matrix and the loading matrix in the principal space corresponding to the largest singular values, whereas the residual matrix $\tilde{X}$ can be further decomposed into the product $\tilde{T}{\tilde{P}}^{T}$, if desired.

The decomposition is made such that $TT^{T}$ is orthogonal and $PP^{T}$ is orthogonal too. The matrix ***E*** denotes the residual portion of ***X***. The columns of the matrix ***P*** are the eigenvectors corresponding to the chosen number (lower than *m*) of *l* largest eigenvalues of the covariance matrix ***S***, and the columns of $\tilde{P}$ are the eigenvectors corresponding to (*m*-*l*) eigenvalues, which are the smallest ones. This approach guarantees that no other orthogonal expansion of *l* components that capture more variations of data exists.

Determination of the number *l* of principal components usually influences the sensitivity of fault detection with the use of the PCA method. To significantly reduce data dimensionality, the number *l* should be as small as possible (i.e., *l* << *m*), but on the other hand, as much data variability as possible should be retained (which is the argument for the choice of bigger values of *l*). An intuitively plausible general approach, proposed in [24], considers the cumulative percentage of total variation that the selected PCs contribute:

(9) $${cr}_{m}=100\cdot \frac{\sum_{k=1}^{l}\lambda_{k}}{\sum_{k=1}^{m}\lambda_{k}}\;\left[\%\right]$$

It turns out in practice that when the accumulated contribution rate ${cr}_{m}$ is above 85% (or between 80% and 90%), the first *l* principal components could sufficiently reflect the main information about the system [26]. Another method of determination involves the *scree graph*, which is a plot of $l_{k}$ against *k* (*k* = 1, ..., *m*). Looking at the plot, we can decide at which value of *k* the slopes of lines joining the plotted points are “steep” to the left of *k*, and “not steep” to the right [24]. This value of *k*, which corresponds to an “elbow” in the graph, is then taken to be the number of retained principal components, *l*.

### 3.2. Multiway Principal Component Analysis

Multiway principal component analysis (MPCA) is an extension of principal component analysis for three-dimensional data. It is performed by initially unfolding the three-dimensional data array to a two-dimensional matrix and then by the application of principal component analysis to the resulting two-dimensional matrix. This approach is particularly useful to process batch process data, which typically comprises measurement of *J* process variables (*j* = 1, 2, ..., *J*) recorded at regular time intervals (*k* = 1, 2, ..., *K*) throughout the batch run. A similar data segment is collected for a number of *I* batch runs (*i* = 1, 2, ..., *I*), so the information about the process can be organized into a three-dimensional data array, ***X***.

**Figure 1:**
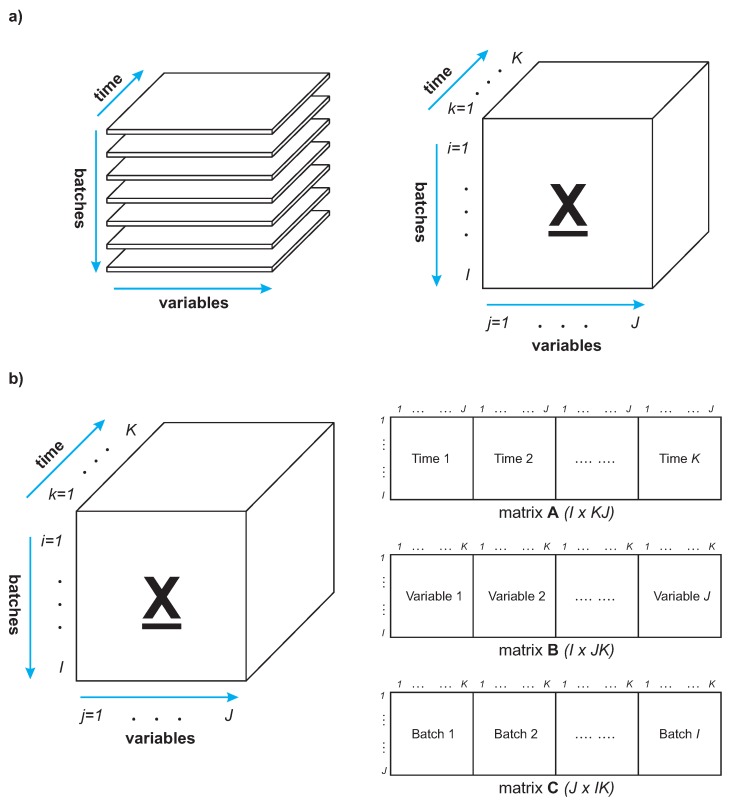
Graphical representation of methods to unfold a three-dimensional data matrix: (**a**) Three-dimensional data matrix of a batch process. (**b**) Unfolding methods of a three-dimensional data matrix.

There are three possible methods to unfold the data matrix ***X*** to perform the ordinary PCA decomposition [27], which is illustrated in Figure 1. In each case, the direction of one axis is preserved and the directions of the other two axes are transposed, resulting in three two-dimensional matrices: ***A*** (*I* × *KJ*), ***B*** (*I* × *JK*) and ***C*** (*J* × *IK*). In the construction of matrices ***A*** and ***C***, measurements of process variables logged at the same time are kept together for all batch runs. For the matrix ***B***, measurements of an individual variable during the batch duration are kept together for all batch runs. The approach introduced in [28] (and used in the experiments described in this paper) is to unfold the three-dimensional data matrix to a two-dimensional data matrix by preserving the direction of the batches, which results in a two-dimensional data matrix ***A*** (see Figure 1).

The objective of MPCA is to decompose the two-dimensional unfolded matrix ***A*** (using standard PCA) into a summation of the product of the score vectors ($t_{l}$) and the loading matrices ($P_{l}$), plus a residual matrix ***E*** that is minimized in a least squares sense:

(10) $$A_{IxKJ}=\overset{L}{\underset{l=1}{\sum }}t_{l}⊗P_{l}+E=\overset{L}{\underset{l=1}{\sum }}t_{l}p_{l}^{T}+E$$

where *L* is the number of principal components.

A score vector $t_{l}$ represents the relationship among *I* batches (each element of the score vector expresses the projection of the *l*-th batch onto the reduced space), while the loading matrix $P_{l}$ is related to the sensor readings (*j*) and their time variations (*k*). Loading matrices ${\left\{P_{l}\right\}}_{l=1}^{L}$ store all the information about how the sensor readings deviate from their mean values at each sample.

The concept of application of the MPCA to process monitoring consists of the use of correct batch data to build a model (of a substantially reduced dimensionality) which describes the normal behavior of the process. A new batch (the test batch) is then monitored by comparing the projected data in the reduced space with the corresponding projections from normal-batch data. A preliminary classification of a new batch $X_{n}$ (*K* × *J*) can be tested for any unusual process behavior by obtaining its predicted t-scores and residuals [29]. If a new batch is similar to a specific class in the data used for the MPCA model development, its $t_{test}$ scores will be located near the origin of the reduced L-dimensional space and the residual should be small. The distance of a test batch from the origin of the reduced space can be measured by the Hotelling’s $T^{2}$ and the squared prediction error (SPE) statistics, described below. The similarity among batches can be also compared by plotting their scores (the projection of each batch in the reduced variable space) and evaluating their location against the confidence region, which (in some sense) bounds the area of the normal process operation.

### 3.3. Hotelling’s $T^{2}$ and Squared Prediction Error (SPE or Q) Statistics

For on-line process monitoring based on PCA, a general approach for detecting an abnormal status employs the $T^{2}$ and *SPE* (*Q*) statistics for the loading vectors retained in the PCA model [20]. The $T^{2}$ statistics measures the variations in the score space and can detect most of the faults that produce large mean shifts in the measurement variables. The $T^{2}$ statistics can be computed by:

(11) $$T^{2}=x^{T}P\Sigma_{l}^{-2}P^{T}x$$

where $\Sigma_{l}$ contains the first *l* rows and columns of the matrix Σ, and **x** is the observation vector from the data set.

The appropriate threshold for $T^{2}$ statistics, based on the level of significance α, can be determined by assuming that the observations are randomly sampled from a multivariate normal distribution and the $T^{2}$ statistic follows the $\chi^{2}$ distribution with *m* degrees of freedom [22]. The threshold of the $T^{2}$ statistics can be then determined as defined below:

(12) $$T_{\alpha }^{2}=\frac{\left[(n^{2}-1)m\right]}{\left[n(n-m)\right]}F_{\alpha }(m,n-m)$$

where $F_{\alpha }$*(m, n-m)* is the value of *F*-distribution at the significance level of α with *m* and *(n-m)* degrees of freedom.

The squared prediction error (*SPE* or *Q* statistics) measures the amount of variation not captured by the PCA model, which may be considered as the lack of fit of the PCA model to the data. If we decompose a new measurement vector $x_{k+1}\in R^{n}$ into two parts, following (Equation (8)):

(13) $$x_{k+1}\;=\;{\hat{x}}_{k+1}+{\tilde{x}}_{k+1}\;=\;{\hat{x}}_{k+1}+{\tilde{e}}_{k+1}$$

so the first component of the above sum is explained by the PCA model, while the second one is the prediction error [30]. The *Q* statistics is then defined on the residual vector ${\tilde{x}}_{k+1}$:

(14) $$Q\;=\;{||e_{k+1}||}^{2}={\tilde{x}}_{k+1}^{T}{\tilde{x}}_{k+1},\;\;\;\;where\;\;\;\;\tilde{x}=(I-PP^{T})x$$

i.e., on the portion of the measurement space which corresponds to the lowest *(m-l)* singular values.

The *Q* statistics can be monitored by using the threshold value, computed as:

(15) $$Q_{\alpha }\;=\;\theta_{1}{\left[\frac{C_{\alpha }\sqrt{2\theta_{2}h_{0}^{2}}}{\theta_{1}}+1+\;\frac{\theta_{2}h_{0}(h_{0}-1)}{\theta_{1}^{2}}\right]}^{\frac{1}{h_{0}}}$$

where

(16) $$\theta_{i}\;=\overset{m}{\underset{j=l+1}{\sum }}\lambda_{j}^{i}\;\;\;\;\;\;i=1,\;2,\;3$$

(17) $$h_{0}\;=\;1-\frac{2\theta_{1}\theta_{3}}{3\theta_{2}^{2}}$$

and $c_{\alpha }$ is the normal deviate corresponding to the upper *(1−α)* percentile.

Any value of $T^{2}$ and *Q* statistics that exceeds the threshold values defined by Equations (12) and (15) indicates abnormal (faulty) conditions of the process under monitoring.

## 4. Boiler Water System in Elektrocieplownia Bialystok

**Figure 2:**
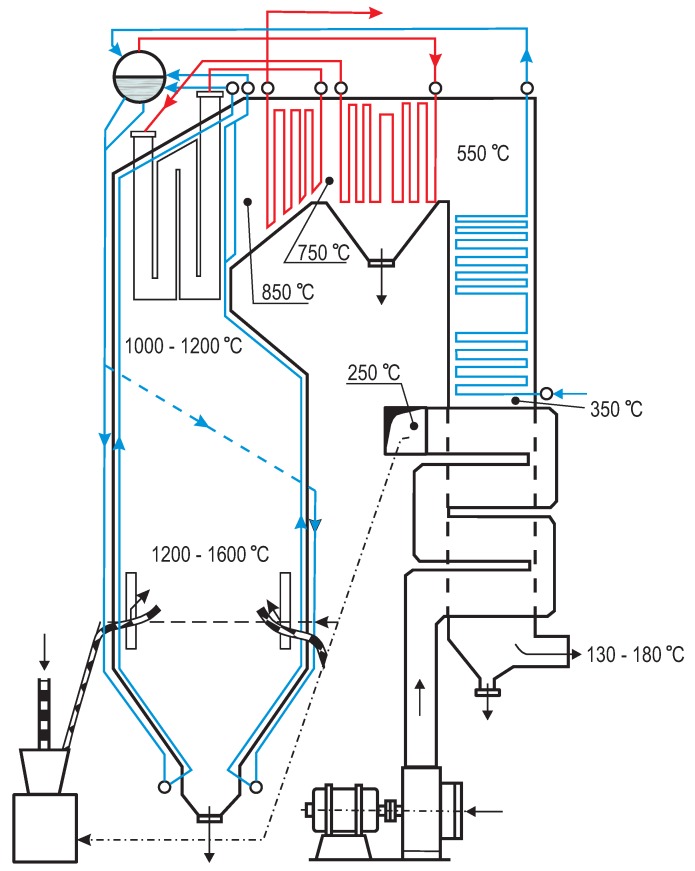
The schematic view of the OP-230 boiler [31].

The OP-230 one-drum and two-pass boiler with a natural water circulation, schematically presented in Figure 2, is a part of the BC-50 thermal unit. The unit, manufactured by RAFAKO Ltd. (Raciborz, Poland), is 27 m high, 7.5 m deep and 8.4 m wide. Operating between 25 MWe and 55 MWe, it can provide maximum continuous output of 230 t of steam per hour and has a back-pressure extraction turbine for district heating. The OP-230 is tangentially fired with pulverized bituminous coal and equipped with four burner columns (six levels of swirl burners in each of them) installed in each of four corners. The main elements of the OP-230 boiler are: the drum; combustion chamber water walls; the steam reheater and superheater; two attemperators; one economizer; two rotary air heaters; and a supporting structure with a casing and platforms.

The steam from the drum is supplied to the first stage of the convection superheater in the first pass, followed by the first steam attemperator. Then, the steam flows to a platen superheater (the second stage), the second steam attemperator, the steam superheater of the third stage and the outlet collector. The air is supplied to the fans both from inside and outside of the boiler room. A tube type economizer is located in the second pass of the boiler. The swirl burners generate short and wide flame, yielding corrosion problems on the walls and elements inside the furnace. The OP-230 boiler is equipped with antiexplosion protection.

In the period 2007–2009, Honeywell’s distributed control system (DCS) “Experion Process Knowledge System” was developed and installed in Elektrocieplownia Bialystok. As a part of that highly integrated process control and management system, the OP-230 boiler was equipped with 12 automatic control subsystems, which enable stabilization of the main process variables, and proper running of fuel combustion and the steam generation process. The “Experion PKS” has the following main control and safety functions: burner/boiler management, process safeguarding and emergency shutdown, turbine and compressor safeguarding, fire and gas detection and pipeline monitoring. The DCS system provides the operators with complete real-time information about current operating conditions of the process and its individual elements and about certain process faults and failures.

In the boiler section of the heat generation process, the following control subsystems enable it to maintain a stable desired operating point:

- Pressure in the combustion chamber (furnace draft);
- Air flow, supplying the burners;
- Air flow to OFA nozzle;
- Air temperature after the steam air heater;
- Contents of O_2_ in the exhaust fumes;
- Contents of NO*_x_* in the exhaust fumes;
- Temperature of the air-pulverized fuel mixture after the mills (four control loops);
- Air flow delivered to the mills (four control loops);
- Boiler load;
- Steam temperature at the boiler outlet;
- Steam pressure at the boiler outlet;
- Drum water level.

As commonly happens in industrial practice, even sophisticated fail safe control systems do not eliminate the failures of process devices or the measurement and control equipment. In the last few years, about 30 major failures per year occurred in the power generation units, which caused the unscheduled shutdowns of the plant. In that number, about one third were failures caused by leakages located in the boiler (see Table 1, which shows the reasons for the shutdowns of the units). The inspections performed during repair confirmed that the common boiler fault was the pipeline leak in the riser and downcomer sections, mostly due to aging and thermal stress. The faults of this kind are rather difficult to detect at their early stage by the diagnostic systems or by the process operator during usual on-line monitoring of the plant [32].

## 5. Application of the MPCA to Detection of Boiler Pipeline Leaks in Elektrocieplownia Bialystok

This section describes the application of the MPCA method to early detection of leaks in a pipeline system of a steam boiler in Elektrocieplownia Bialystok. In a series of numerical experiments, we studied several episodes of tube cracks, which caused emergency shutdowns of the boiler during the period 2011–2016. We utilized the process variables which are measured for control purposes and stored in a process database. We could also access the protocols from the inspection of the pipeline system during its repair, which contained detailed description of the failure, together with its photographic documentation. Unfortunately, any statistical conclusions about the efficiency or the accuracy of the presented method cannot be made from our experiments, because the number of leakages which occurred within the above period was relatively small. That is why we discuss two cases which present the ability of the MPCA method to detect leaks requiring unplanned safety shutdowns of the boiler, several hours (or even days) earlier than they were noticed by the personnel.

In an industrial steam boiler, it is very hard to determine the precise moment when a leak arises, and the period from creation of the miniature hole in a pipeline to the moment the leak reaches the size when the operating staff can notice clear symptoms of the failure. Additionally, the length of the pipeline section of a boiler amounts to several dozens of meters, and the cracks may appear in random locations, so, typically, the development of such faults varies with time and influences process variables in a very different way. In most cases, a single leak causes cracks in neighboring tubes, which can be regarded as failure propagation and multiplication. The algorithm of tube leak detection must utilize measurements of several process variables and correlate them with typical fault patterns.

### 5.1. Data Analysis and Preprocessing

In the integrated Honeywell’s distributed control system (DCS) “Experion Process Knowledge System,” installed in the OP-230 boiler, 37 process variables are directly measured and recorded in the process database. After careful analysis of historical data (recorded during previous leakages and during normal operating conditions), and after discussions with process operators, we selected 12 variables, potentially most sensitive to leakage development. In that number, there are both the quantities measured at the input and at the output of the heating process (water and air flow at the boiler input, steam flow at the boiler output), and some other variables measured in specific locations in the plant (two temperatures of steam, five temperatures of fumes, lift in the hearth chamber and O${}_{2}$ concentration). As the changes of the course of those variables in the period preceding the boiler shutdown were significant, we decided to use them (as well as their different subsets) as the inputs to the MPCA algorithm, studied in our leak detection experiments.

**Figure 3:**
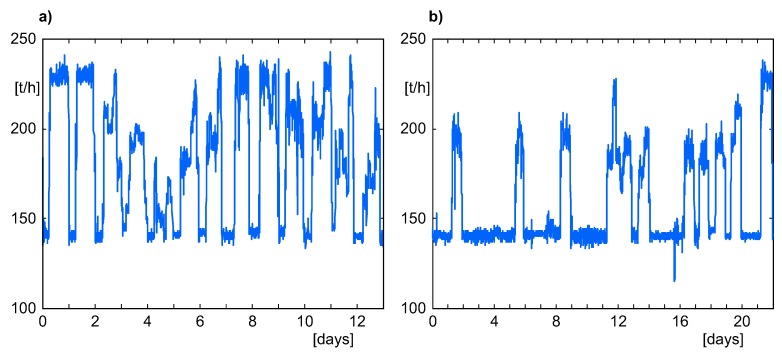
Daily variability of the steam boiler load for time periods described in Section 5.3: (**a**) 04.12.2011. (**b**) 13.01.2014.

It should be emphasized that the power or steam generation processes are rather far from being typical batches; they rather belong to the group of nonlinear, nonstationary processes. The boiler operating point (which obviously influences daily profiles of steam quantities) is set manually by the operator, due to seasonal and daily demands, the loads of other boilers cooperating with the considered one and (to some extent) the abilities of the thermal-electrical power plant to accumulate energy. That is why (e.g., in a monthly period) daily profiles of steam production sometimes vary substantially, depending on variability of demands on co-generated heat and electricity. That is clearly visible in Figure 3, where the profiles of steam load in the periods just before two shutdowns described as the case studies, which took place on 04.12.2011 (Figure 3a) and 13.01.2014 (Figure 3b), are presented. However some similarities between daily profiles of process variables exist which justified our trials to employ the MPCA method to detect symptoms of pipeline leakages. It turned out (this will be discussed in detail below) that in such difficult conditions, the MPCA method was able to detect developing leakages in advance, providing the process operator sufficient time to undertake appropriate actions.

For the purposes of the distributed control system, all the process variables are measured and recorded in the historical database with the sampling period of 2 s. Such a sampling interval is definitely too short, regarding the approximate dynamics of the tube cracking process and the functioning of the system which would support leak diagnostics. That is why the original signal samples were aggregated by averaging them in the period of 2 min (i.e., the average was computed from 60 originally recorded samples). In some numerical experiments the longer interval of 5 min has been also used, but the shorter averaging period gave better results of leak detection. The averaging may be also considered as the very simple operation of signal filtration to remove fast fluctuations of the process variables, irrelevant from the point of view of the diagnostic procedure.

### 5.2. Solution of the Boiler Leak Detection Problem with the Use of Multiway PCA

The experimental approach to leak detection described in this paper had the following two steps: the design (learning) phase and the monitoring (testing) phase. In the design phase, the set of historical data was used to develop the MPCA model of the “healthy” pipeline system of the boiler. Data used for model development represent the segments of 12 process variables mentioned above (or their selected subset), collected in the same time period. The length of the data segment to be split into batches should be sufficiently long; typically, 20–30 batches have been used to develop the MPCA models in the works reported in literature (e.g., [33]). Unfortunately, the periods of the steady operation of the boiler in Elektrocieplownia Bialystok were not as long (mostly due to plant shutdowns from other reasons or very large changes in its operating policy), so the number of 12–25 batches was used in our experiments. The data matrix (containing one process variable in a column) was then normalized by removing the mean value and dividing by a variance of each column.

For each variable, its measurements from several consecutive days were split into daily data segments to create batches; each batch started at 0:00 and ended at 23:58, which enabled the performance of leakage detection only once a day, when the full batch was completed. This problem arises in general, when MPCA monitors a new batch, because at any point in time, only the data up to then are available and nothing is known about the remainder of the batch [34]. In order to calculate the score vectors for the present batch, the missing data would have to be filled with one of several methods suggested in [28]. Usually, two methods are used to solve this problem in practice: the first is to put zeros in the vector for all remaining missing batches, and the second approach fills in the future data with the current observed value that has been normalized. We assumed that at this stage of the experiments, a daily frequency of diagnostics was sufficient; however, the approach where the batch start and end moments are changeable, according to the moment when the detection task is performed, seems to be quite promising. For example, if the fault detection process is performed at 08:00, all the batches are created from long data, starting each segment at 08:00 and ending it at 07:58 in the next day. In that way, considering the fact that the steam generation is not a typical batch process, we can utilize the sliding window to arrange the data split into batches of the same length, created from real measurement data. Several experiments employing such an approach (not reported here) gave reasonable results of early detection of leaks.

Based on the three-dimensional data unfolding method proposed in [28], the principal components model has been established, and then the principal components contribution rate has been calculated [35]. For the simplicity of representation of the “fault trajectory” (described below), the reduced principal component subspace of three dimensions has been chosen. Such a reduced dimensionality is not in full agreement with the guidelines given in [24,26], as for the two cases described below and the set of 12 original process variables decomposed by PCA, three principal components gave the accumulated contribution rates c${}_{rm}$ (expressed by Equation (9)) of 64% and 75%, respectively. However there were not substantial qualitative improvements of the detection results for 4- and 5-dimensional principal component subspace, where the values of c${}_{rm}$ were in the ranges 76–82% and 84–86%, respectively.

**Figure 4:**
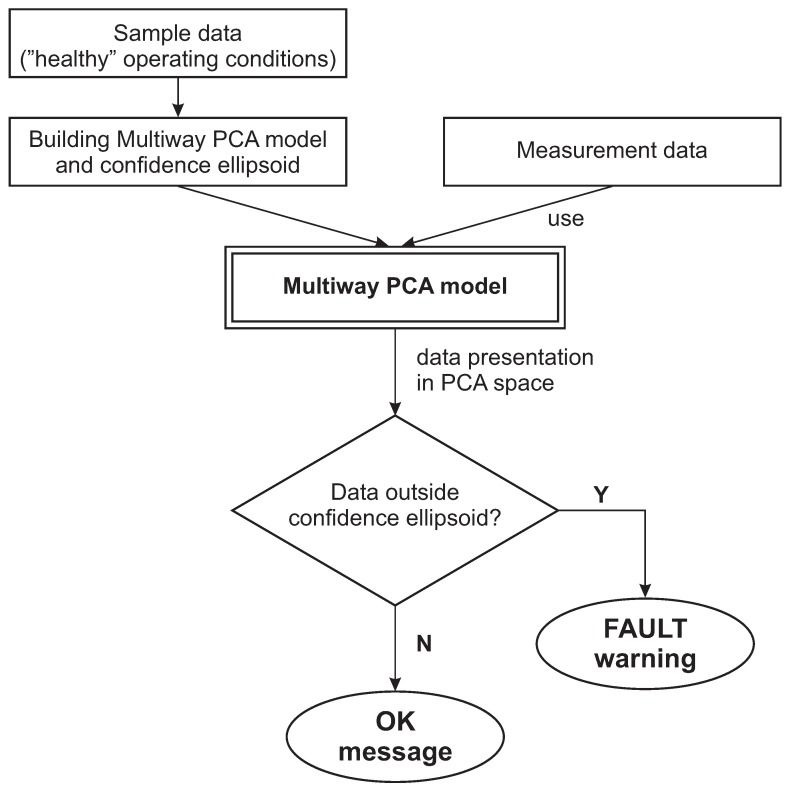
Block diagram of the MPCA-based pipeline leak detection.

The points representing normal batches, which were used to develop the MPCA model, were then mapped on the coordinate system created from three principal components (scores) of the largest contribution. The region containing 95% of such mapped points may be bounded by an ellipsoid, which is considered the confidence region (control limit). The location of the center and the size of the ellipsoid represent the mean and the covariance of the mapped data used for development of the MPCA model. The mapped data points located inside the ellipsoid represent the “healthy” operating conditions of the boiler pipeline system. During the monitoring phase, the newly collected batch (representing the last daily segment of process variables) is normalized and mapped to the principal components coordinate system. The location outside the confidence ellipsoid is the indicator of possible fault appearing in the pipeline system. The finding may be also supported by the results of comparison of the values of Hotelling’s *T*${}^{2}$ (Equation (11)) and SPE (Q) statistics (Equation (14)) with their thresholds given by Equations (12) and (15), respectively. The block diagram of the detection scheme is presented in Figure 4.

### 5.3. Two Case Studies

In our experiments, we used data recorded in the period 2011–2016, after substantial renovation of the boiler; i.e., replacement of some sections of pipelines due to creeping degradation of pipelines and an increasing number of leaks in previous years. To illustrate the ability of the MPCA method to detect leaks in the pipeline system of the boiler, we have chosen two cases of documented faults; one of them required unplanned shutdown of the boiler. The process was shut down shortly after the personnel undoubtedly determined the cracks (mostly on the basis of acoustic examination), and during the inspection of the pipeline system after the shutdown, the crack was confirmed. In the first case described below the personnel did not notice any symptoms of leakage. In the second case, the first symptoms of irregularities in the measured process variables could be observed by the operator less than 10 h before such an auditory confirmation of the fault. As it will be shown below, using the approach based on the MPCA decomposition, the leaks could be clearly detected significantly earlier.

In both experiments presented below, at first, the full set of 12 process variables (mentioned above) was used to prepare the MPCA model of the “healthy” system, with the principal component space reduced to the three most significant components. Then, we looked for the subset of process variables (i.e., containing the lower number of process variables) which could be employed as the minimal indicator of leakages. Successfully, five such variables were found and used in the case studies reported below. In the experiments, we did not take advantage of prior knowledge gotten from the operator—whether the data represented a leak and when the leak had taken place, or whether the plant should be shut down as a result of the leak. The detection scheme followed the flowchart presented in Figure 4, which enabled us to construct the “fault trajectory” in the principal component space, which at least four days before the boiler shutdown, goes outside the confidence ellipsoid. Additionally, the Hotelling’s T${}^{2}$ and SPE (Q) statistics (not discussed here) confirmed the symptoms of pipe leaks detected by the algorithm.

#### 5.3.1. Case 1—Boiler Operation without Clear Symptoms of a Failure

At about 00:05 on 4th December 2011 the boiler was shut down according to schedule, because the heat accumulation system had to be discharged. During the usual inspection, at about 6 o’clock, the personnel found the crack of a stub pipe in the left side of a chamber. In the period of the process operation the arising fault did not give any symptoms of the failure which could be noticed by the process operator, based on the measurements of process variables. There were no noticeable differences in the observed level of process disturbances, compared to the period of the “healthy” operation of the boiler.

**Figure 5:**
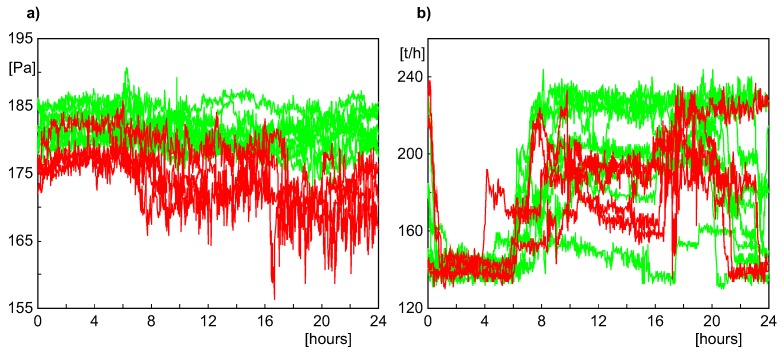
Two process variables from the period from 17th November 2011 to 4th December 2011, after splitting into one-day batches: (**a**) Lift in the hearth chamber. (**b**) Flow of the water feeding the boiler.

We performed a series of numerical experiments to check the ability of the MPCA method to early detect the leakage in the above case. We attempted to get a clear fault warning from the MPCA model at least four days before the boiler shutdown. In the simulations, several subsets of variables, selected from the set of 12 process variables (mentioned above) were employed to create the MPCA model and confidence ellipsoid. The main difficulty in building the MPCA model consisted of an extremely small number of batches and the huge variability of the shapes of process variables. The period of an uninterrupted and relatively stable operation of the boiler was very short and lasted from 17th November 2011 to 4th December 2011, so according to the experiment setup, only data from the last two weeks of November were used to create the MPCA model of a “healthy” system. The graphs showing variability of two process variables (the lift in the hearth chamber and the flow of the water feeding the boiler) split into one-day batches are presented in Figure 5. The batches used for building the MPCA model are shown in thin green lines, while those considered faulty (and employed to check the detection ability of the model) are shown as thick red lines. It can be easily realized that the theoretical assumptions about repetitiveness of the batches (required by the MPCA method) are hardly fulfilled, and the shapes of individual variables representing faulty conditions do not differ essentially from several “healthy” batches.

**Figure 6:**
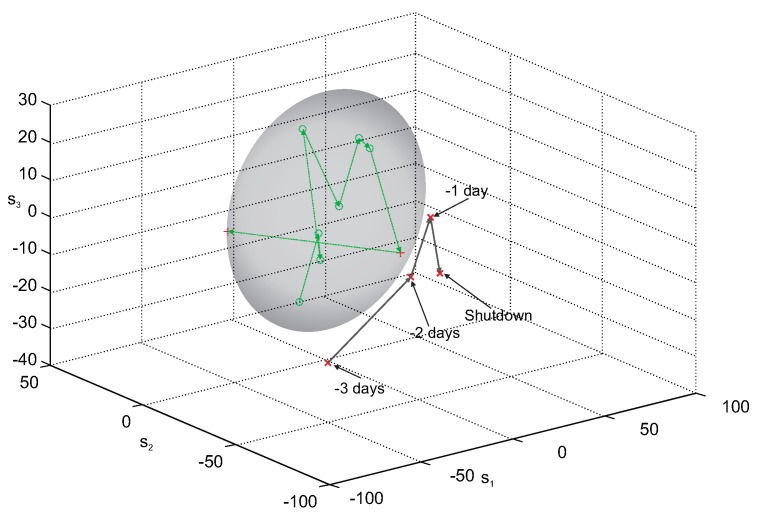
Fault trajectory in the 3-dimensional principal components’ space in the three days period before the shutdown—MPCA decomposition method (pipes damaged on 4th December 2011).

Figure 6 presents the results of leakage detection with the use of the MPCA model created from the experimentally selected set of five process variables, decomposed by MPCA. The MPCA model in the principal component (PC) space spanned by the largest three PCs, was used to obtain the confidence ellipsoid, which bounds the area corresponding to “healthy” data. The circles in Figure 6 represent the points (batches) which lay within the confidence ellipsoid, while all the crosses correspond to batches placed outside the ellipsoid. The thin green dotted line represents the “healthy” process trajectory, constructed from the representation of the consecutive batches, projected on the reduced three-dimensional PC space. The graphite solid line represents the fault trajectory; i.e., the representation of the last four batches (which precede the moment of the boiler shutdown) in the PC space.

As it can be seen, despite the unfavorable conditions for building the MPCA model, the fault trajectory is fully located outside the confidence ellipsoid. Additionally, the representation of the last batch, which has been used to create the MPCA model, is located close to the border of the “healthy” region, which can be the evidence of the first symptoms of the tube crack. Such a trajectory may give the process operator clear information about disadvantageous phenomena occurring in the pipeline system, which cannot be obtained from the observation of direct recordings of process variables. It should be emphasized that in the case of the fault described here, the process operator did not notice any symptoms of an arising leakage.

#### 5.3.2. Case 2—Emergency Shutdown of a Boiler

**Figure 7:**
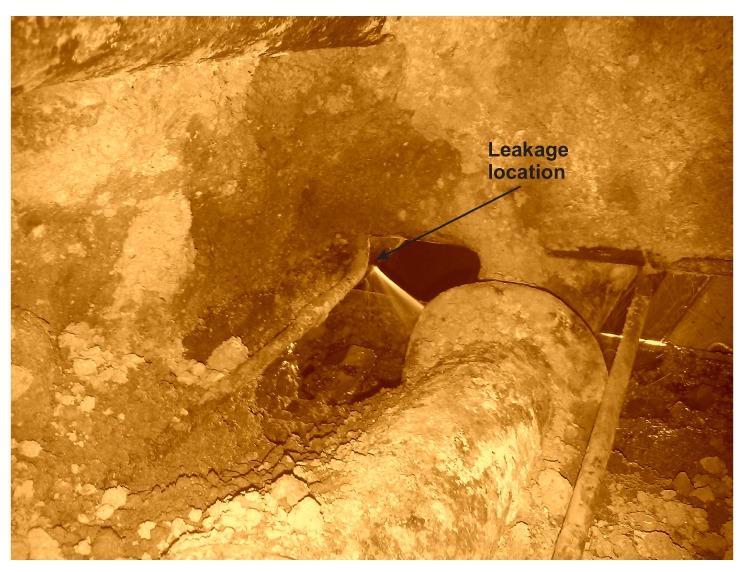
Leakage location, pipes damaged on 13th January 2014.

The first symptoms of the failure were noticed by the process operator at about 1 o’clock in the night on 13th January 2014, on the basis of personnel reports from the routine round and on the observation of small disturbances in measurements of process variables. The leak was then increasing quite fast, but the boiler was kept working until 9:46 on 13th January 2014, when the substitute boiler was started. The inspection of the lower area of the collector chamber, performed during the boiler repair, revealed the crack presented in Figure 7.

**Figure 8:**
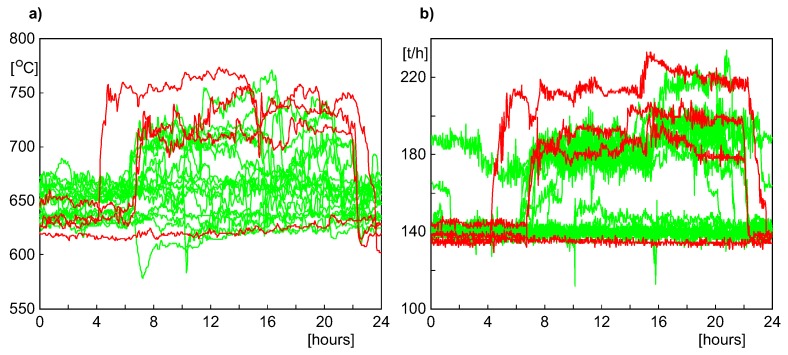
Variability of two process variables in the period from 22nd December 2013 to 13th January 2014, after split into one-day batches: (**a**) Temperature of fumes in the left channel. (**b**) Flow of water feeding the boiler.

In the numerical experiments, we used the data segment recorded between 22nd December 2013 and 13th January 2014 to build the MPCA model and check its ability to detect the leakage three days earlier than the emergency shutdown. In that case, the number of batches was bigger than in the previous case, as the period of an uninterrupted and relatively stable operation of the boiler (which could be considered as the “healthy” period) lasted for 19 days. However, the variability of the shapes of process variables was still significant, as is shown in Figure 8 for two process variables (the temperature of fumes in the left channel and the flow of water feeding the boiler). As in Figure 5, the batches used for building the MPCA model are shown in green color, while those considered as faulty (and employed to check the detection ability of the model) are shown as thick red lines.

**Figure 9:**
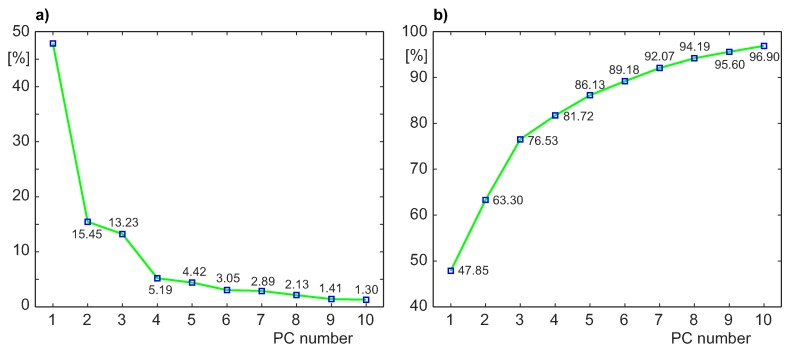
Explanation of the selection of the principal components which create the MPCA model: (**a**) Scree plot—percent of variance captured by the first 10 principal components. (**b**) Accumulated contribution rate *cr${}_{m}$* for the first 10 principal components.

Figure 9 shows the scree plot of the percent of data variance captured by the first 10 principal components and the accumulated contribution rate ${cr}_{m}$ (Equation (9)). As can be seen, retaining three principal components preserves about 76.5% of data variance. Such a value is slightly below the threshold of 80% of preserved variability, recommended in [24,26]; however, a kind of an “elbow” is visible on the scree graph after the 3rd principal component. The plots presented in Figure 9, together with the fact that the confidence ellipsoid and the “fault trajectory” should be presented to the operator in a comprehensible manner, confirm our choice of three Principal Components.

**Figure 10:**
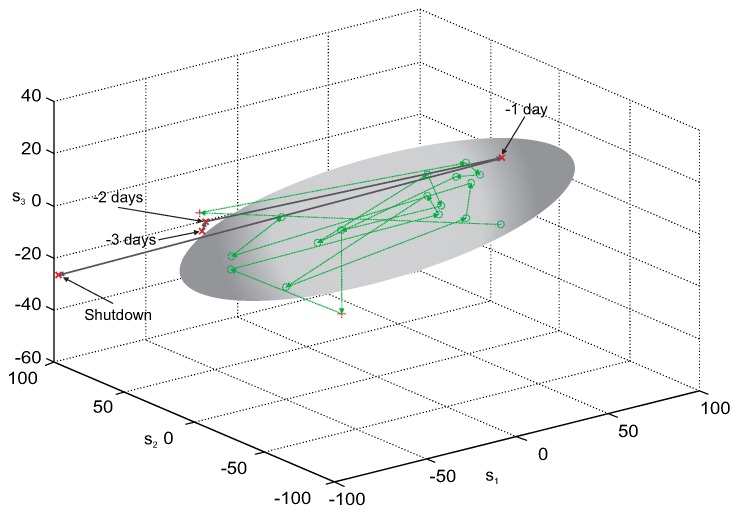
Fault trajectory for leakage location—pipes damaged on 13th January 2014.

Figure 10 presents the results of leakage detection with the use of the MPCA model created from the subset of five variables, selected experimentally from the full set of 12 process variables. As in *Case 1*, the MPCA model in the principal component (PC) space spanning the largest three PCs, was used to obtain the confidence ellipsoid. The circles in Figure 10 represent the points (batches) which lay within the confidence ellipsoid, bounding the area occupied by “healthy” data. The thin green dotted line represents the “healthy” process trajectory, constructed from the representation of the consecutive batches, projected on the reduced three-dimensional PC space. The crosses correspond to batches placed outside the ellipsoid, and the graphite thick solid line represents the fault trajectory. The fault trajectory is the representation of the last four batches (which precede the moment of the boiler shutdown) in the PC space.

The MPCA model, even if it was built from data with substantial variability of process operating conditions, was able to detect arising fault much earlier than it was done by the process operator. The fault trajectory, presented in Figure 10, is fully located outside the confidence ellipsoid, so it may give the process operator important, unambiguous information about arising leakage.

## 6. Conclusions

The studies presented in this paper confirmed that the MPCA method is a useful tool for early detection of a specific class of faults; i.e., the leakages in the pipeline system of a steam boiler. All the numerical experiments reported in the paper have been performed using real data obtained from an industrial plant. The processes of heat and electricity generation in a thermal-electrical power plant are not typical batch processes, where the same (or at least very similar) raw materials and operating conditions yield periodically repeatable shapes of process variables. Production of heat and electricity, strongly associated each with other, vary according to seasonal, weakly and daily demands of customers. Various characteristics of biomass (which is used as fuel for this type of thermal units) also cause substantial differences between daily profiles of process variables. Nevertheless, even rough similarity of batches enabled us to create the MPCA model, which provided the process operator with important information about a growing leakage in a pipeline system.

Two case studies described in the paper were not the only examples of the ability of MPCA to early detect the leaks in the boiler pipeline system. We analyzed 23 cases of emergency shutdowns of the boiler, caused by leakages occurring in the period 2011–2016. The number of such faults in that period was bigger (see Table 1), but some of the cases had to be excluded from analysis, due to very short time of stable operation before the shutdown (e.g., caused by earlier shutdowns or operating policy, changing distribution of steam production between boilers) or enormous variability of shapes of process variables. As it can be seen from Table 2, only one of 23 leakages (i.e., about 4.3%) was detected by our method not earlier than 24 h before the shutdown. In all the cases analyzed in our experiments, the MPCA method could detect the abnormal behavior of the process 2–5 days before the shutdown. That means that the last 2–5 points representing consecutive batches in the PC space (as in Figure 6 and Figure 10) were located outside the confidence ellipsoids. In most cases, the reaction of the solution presented above preceded the moment when the fault symptoms were noticed by the personnel.

In our opinion, the proposed MPCA approach, or the approach based on a standard PCA model studied in [32], can replace the necessity of the construction and identification of the process model, which is time consuming and relatively expensive, so for those reasons not applicable in Elektrocieplownia Bialystok. The method described in the paper can effectively extract information of growing leakages, so the implementation of our detection approach may be the important element of an on-line system, which would lead to significant improvement in safety and maintenance of industrial boilers in Elektrocieplownia Bialystok. The early warning, based on the MPCA model, can be given for plant operators in a very simple and comprehensible manner, as the presentation and analysis of the fault trajectory in the “confidence space,” which represents the feasible area occupied by the values corresponding to process variables for the “healthy” system. It is also worth noting that the concept of the future advisory structure for early leak detection consists of full cooperation with the existing distributed control system.

We are also aware of some limitations of the proposed approach, as well as simplifications applied to our algorithm. First—the impact of data quality or arising faults in the measuring paths on generated diagnoses should be carefully studied before any attempt to implement our algorithm in practice. Additionally—as the starting moment of leakage appearance and the dynamics of its development are unknown and undetectable, it is possible that data used for building the MPCA model (considered as “healthy”) may contain the symptoms of developing leakage. It is intuitively feasible that such a situation somehow influences detection accuracy; however, the loss of accuracy is imposible to be estimated and the poor quality of data influences any data-driven plant model. Despite the above objections, the MPCA study presented in this paper (as well as previously reported PCA-based approach) may provide a useful tool supporting decisions of plant operators.

## Figures and Tables

**Table 1:** Reasons for shutdown of power generating units in the consecutive years.

Reason of the Block Shutdown—Year:	2010	2011	2012	2013	2014	2015	2016
Reaction of safety systems (violation of constraints)	11	19	9	15	19	16	11
Staff error	4	3	1	3	2	3	3
Boiler leak	12	10	9	11	7	7	6
Outer failure (e.g., fire, blackout in electrical grid, etc.)	2	3	2	3	1	4	2
***Total number of shutdowns***	***29***	***35***	***21***	***32***	***29***	***30***	***22***

**Table 2:** Performance of the MPCA model in the detection of boiler leakages.

Time Horizon of Leakage Prediction (Number of Days, n):	<= 1	(1; 2]	(2; 3]	(3; 4]	(4; 5]
Number of cases, for which the leakage was predicted	1	2	7	8	5
n days before its detection by the personnel					

## Abbreviations

The following abbreviations are used in this manuscript:

PCA	Principal component analysis
MPCA	Multiway principal component analysis
PC	Principal component
SVM	Support vector machines
ANN	Artificial neural networks
GA	Genetic algorithms
SPE	Squared prediction error
AI	Artificial intelligence
